# Semi-Rigid (Aminomethyl) Piperidine-Based Pentadentate Ligands for Mn(II) Complexation

**DOI:** 10.3390/molecules26195993

**Published:** 2021-10-02

**Authors:** Jonathan Martinelli, Edoardo Callegari, Zsolt Baranyai, Alberto Fraccarollo, Maurizio Cossi, Lorenzo Tei

**Affiliations:** 1 Department of Science and Technological Innovation, Università del Piemonte Orientale, 15121 Alessandria, Italy; jonathan.martinelli@uniupo.it (J.M.); edoardo.callegari@uniupo.it (E.C.); alberto.fraccarollo7@gmail.com (A.F.); maurizio.cossi@uniupo.it (M.C.); 2 Bracco Research Center, Bracco Imaging SpA, 10010 Colleretto Giacosa, Italy; zsolt.baranyai@bracco.com

**Keywords:** polydentate ligands, Mn(II) chelates, thermodynamic stability, NMR relaxometry, computational modeling

## Abstract

Two pentadentate ligands built on the 2-aminomethylpiperidine structure and bearing two tertiary amino and three oxygen donors (three carboxylates in the case of AMPTA and two carboxylates and one phenolate for AMPDA-HB) were developed for Mn(II) complexation. Equilibrium studies on the ligands and the Mn(II) complexes were carried out using pH potentiometry, ^1^H-NMR spectroscopy and UV-vis spectrophotometry. The Mn complexes that were formed by the two ligands were more stable than the Mn complexes of other pentadentate ligands but with a lower pMn than Mn(EDTA) and Mn(CDTA) (pMn for Mn(AMPTA) = 7.89 and for Mn(AMPDA-HB) = 7.07). ^1^H and ^17^O-NMR relaxometric studies showed that the two Mn-complexes were q = 1 with a relaxivity value of 3.3 mM^−1^ s^−1^ for Mn(AMPTA) and 3.4 mM^−1^ s^−1^ for Mn(AMPDA-HB) at 20 MHz and 298 K. Finally, the geometries of the two complexes were optimized at the DFT level, finding an octahedral coordination environment around the Mn^2+^ ion, and MD simulations were performed to monitor the distance between the Mn^2+^ ion and the oxygen of the coordinated water molecule to estimate its residence time, which was in good agreement with that determined using the ^17^O NMR data.

## 1. Introduction

**Figure 1:**
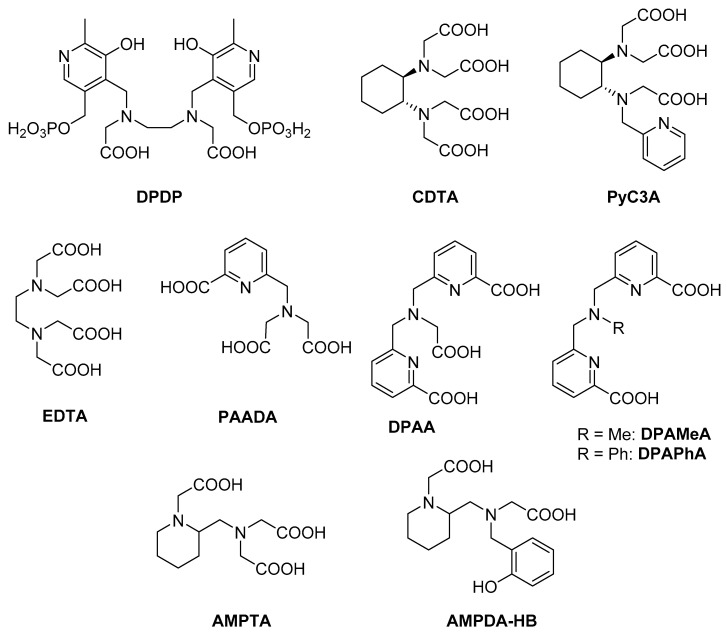
Molecular structures of the ligands mentioned in the text.

Mn^2+^ is an endogenous metal ion that is involved in several biochemical processes and it is present in serum at concentrations of 0.5–1.2 µg/L [1]. Thus, living organisms can efficiently deal with small excess amounts of free metal ions in organs and tissues. Its relative toxicity (LD_50_ = 0.22 mmol/kg) [1] has made it attractive in the last decade as a replacement for gadolinium in contrast agents for magnetic resonance imaging (MRI) since Gd(III) is also toxic in its free form, even in very low amounts, and can be associated with a pathology called nephrogenic systemic fibrosis, which affects patients with compromised renal functionality [2]. Manganese(II) represents a valid alternative to gadolinium(III) in MRI applications because it has a high paramagnetism due to its five unpaired electrons; moreover, it is endowed with a long electronic relaxation time and it can coordinate easily exchangeable water molecules [3,4,5,6,7]. As with Gd(III)-based MRI contrast agents, the relatively high concentrations of Mn(II) that are needed for MRI applications (0.05–0.30 mmol kg^–1^) require the cation to be “caged” by an organic ligand to prevent undesired side reactions in vivo that would compromise its role and cause toxic effects. Such chelators are normally polyamino-polycarboxylates that are capable of strongly binding the metal ion while leaving at least one free coordination position for an exchangeable water molecule: this is an almost compulsory requirement for having a significant contrast enhancement effect with this type of MRI agent. In particular, since Mn(II) typically forms six- or seven-coordinated complexes, using hexa- or pentadentate chelators (Figure 1) was proposed to match the good stability with the presence of at least one binding site available for H_2_O. The only Mn-based MRI contrast agent that was approved for clinical use was Teslascan^®^, where the metal ion is caged by a DPDP (dipyridoxyl diphosphate) ligand (Figure 1) [8]. However, such an agent was recently withdrawn by both the European and US markets because of poor clinical performance and toxicity concerns. Since then, several alternatives have been reported, among which, the Mn-complexes of *cis*-1,4-DO2A and its derivatives (1,4-DO2A = 1,4,7,10-tetraazacyclododecane-1,4-diacetic acid) [9,10], PC2A derivatives (PC2A = tetraazabicyclo[9.3.1]pentadeca-1(15),11,13-triene-3,9-triacetic acid) [11,12], *trans*-CDTA (*trans*-1,2-diaminocyclohexane-*N*,*N*,*N*′,*N*′-tetraacetic acid, Figure 1) [13] and PyC3A (*N*-picolyl-*N*,*N*′,*N*′-*trans*-1,2-cyclohexylenediaminetriacetate, Figure 1) [14] seem to be the most promising for general MRI applications.

Among the many requirements that a metal complex needs to satisfy to be exploited as a contrast agent, in vivo kinetic inertness plays the main role. From this perspective, increasing the rigidity of the chelator may lead to improved stability of the metal complex [13,15]. For example, the source of such rigidity can be a macrocyclic ligand or a cycloalkane/aromatic ring incorporated in the molecular structure of the ligand. In this respect, the presence of a pyridine ring in the chelator structure was recently exploited in a series of penta- or hexadentate non-macrocyclic ligands that were used for the preparation of Mn(II) complexes endowed with enhanced relaxivities, such as PAADA, DPAA and DPAMeA [16,17].

Our group recently reported about two pentadentate ligands based on 2-aminomethylpiperidine (AMP) and bearing acetic and/or hydroxybenzyl pendant arms (namely AMPTA and AMPDA-HB, Figure 1) that were successfully applied for the preparation and test of aluminumfluoride complexes for PET applications [18]. We hypothesized that the presence of the piperidine heterocyclic ring could induce greater rigidity of the Mn(II) complex and therefore greater stability, as occurs in the case of the Mn(CDTA) complex compared to the flexible Mn(EDTA). In addition, the replacement of one acetate arm with a 2-hydroxybenzyl group may increase the binding effectiveness to Mn(II) as the phenolate group is a strong electron donor that can efficiently coordinate various metal ions due to its higher basicity compared to a carboxylate. Moreover, as mentioned above, it is expected that the presence of a rigid aryl ring confers more stability to the corresponding complexes. A few linear or macrocyclic ligands bearing phenol groups were reported in the literature, especially to chelate transition metals, while their applications with lanthanides are rather limited [19,20,21,22].

Thus, in the present work, we describe an improved synthesis of the ligand AMPDA-HB and the detailed investigation on Mn(AMPTA) and Mn(AMPDA-HB) chelates, including ^1^H and ^17^O NMR relaxometric, potentiometric and computational studies.

## 2. Results and Discussion

### 2.1. Synthesis

**Scheme 1:**
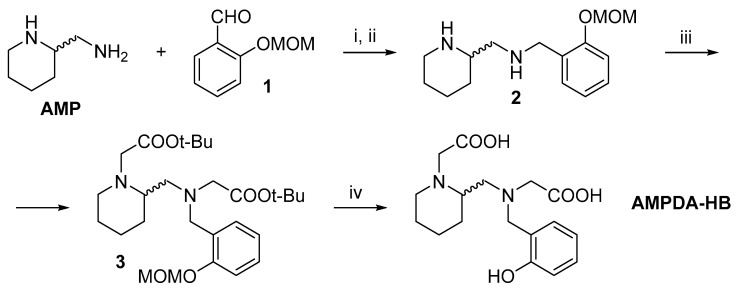
Synthesis of AMPDA-HB from AMP: (i) AcOH, MeOH, rt, 1 h; (ii) NaBH_4_, 0 °C, 2 h; (iii) *tert*-butyl bromoacetate, Na_2_CO_3_, CH_3_CN, rt, on; (iv) TFA, rt, 3 h.

The ligand AMPTA was prepared following the procedure previously reported by our group [18]. For AMPDA-HB, an improved synthesis was developed (Scheme 1), where salicylaldehyde was initially protected at its hydroxyl moiety with a methoxymethyl (MOM) group (**1**) by using methoxymethylchloride [23] and then reacted with racemic 2-AMP via reductive amination to give the intermediate **2**. The remaining available positions on each nitrogen atom were alkylated with *tert*-butyl bromoacetate and the obtained diester **3** was finally treated with trifluoroacetic acid (TFA) to deprotect both the carboxylate and hydroxyl moieties, leading to the intended AMPDA-HB ligand.

Compared with the previous procedure, the slight disadvantage of the further step required (i.e., the MOM protection of salicylaldehyde) was far overcome by the cleaner output that translated into higher yields: keeping the OH group free appeared to have a detrimental effect on both the alkylation (42% yield) and the deprotection (19%) steps, with a total yield of just 2%. In particular, the formation of 2-oxopiperidine bicyclic byproducts was observed due to acid-catalyzed intramolecular aminolysis. Instead, the temporary conversion of the hydroxyl moiety into a methoxymethoxy group allowed for obtaining the diester with an 82% yield and the final compound with a 53% yield, increasing the total yield to 31% relative to the initial amount of AMP. Other strategies, such as the protection of the OH group of salicylaldehyde by using acetate, did not lead to satisfactory results.

**Scheme 2:**
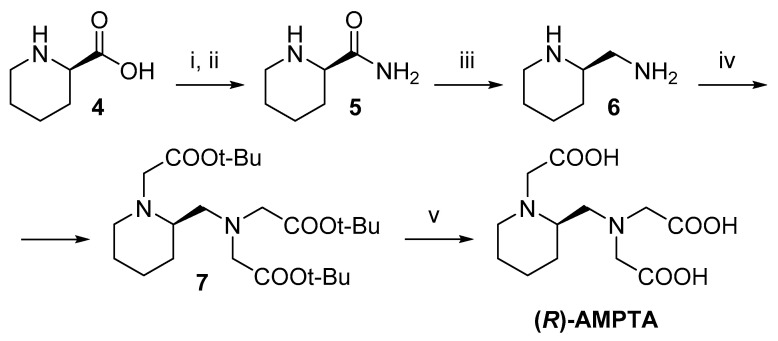
Synthesis of (*R*)-AMPTA: (i) SOCl_2_, MeOH, refl., 2 h; (ii) NH_3_ in MeOH, rt, 2 d; (iii) LiAlH_4_, THF, 0 °C, 3 h; (iv) BrCH_2_COOt-Bu, K_2_CO_3_, CH_3_CN 50 °C, on; (v) TFA, DCM, rt, on.

The *R*-enantiomer of AMPTA was also prepared (Scheme 2), starting from *D*-pipecolinic acid ((*R*)-piperidine-2-carboxylic acid, **4**) via amide **5**, which was obtained according to a reported procedure [24], and was, in turn, reduced to amine **6** with LiAlH_4_. Exhaustive alkylation with *tert*-butyl bromoacetate (**7**), followed by deprotection of the carboxylates under acidic conditions (TFA), led to the intended ligand, whose ESI MS and NMR characterization data were identical to what was reported for the racemic analogs, as expected. 

This chelator was used to prepare the corresponding Mn complex to confirm the correspondence between its relaxometric properties and the results obtained for the racemic mixture.

Complexations were carried out by stirring an equimolar aqueous solution of ligand and manganese (II) chloride at pH 6.5.

### 2.2. Equilibrium Studies

#### 2.2.1. Protonation Equilibria

The protonation constants of H_3_AMPTA and H_2_AMPDA-HB, defined by Equation (1), were determined using pH potentiometry, ^1^H-NMR spectroscopy and UV-vis spectrophotometry. The log*K_i_^H^* values of H_3_AMPTA and H_2_AMPDA-HB are listed in Table 1 and compared with those of pentadentate H_2_DPAMeA and H_2_DPAPhA ligands (Figure 1).

(1) $$K_{i}^{H}=\frac{\left[H_{i}L\right]}{\left[H_{i-1}L][H^{+}\right]}\cdot \cdot \cdot \cdot \cdot \cdot i=1,2\ldots 5$$

**Figure 2:**
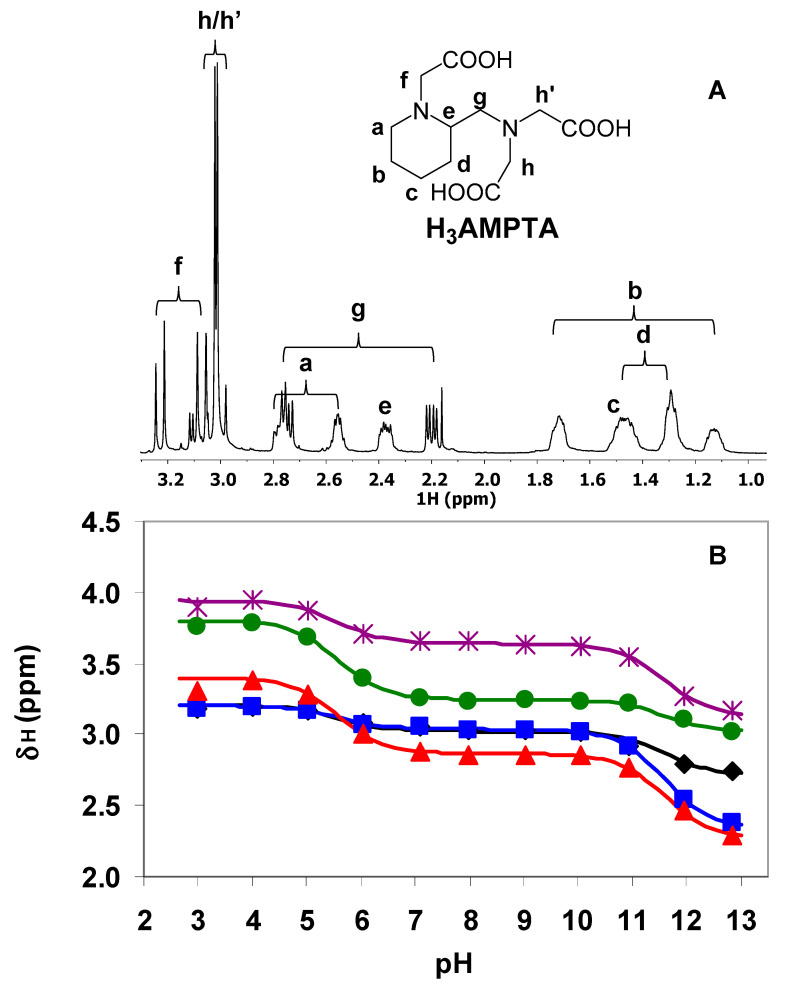
(**A**) ^1^H-NMR spectrum of AMPTA and (**B**) chemical shifts of the different protons as a function of pH. (**A**): pH = 12.84; 500 MHz, D_2_O, 0.15 M NaCl, 25 °C. **B**: a (◆), e (■), g (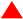), h/h’ (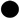) and f (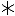), [AMPTA] = 0.01 M, 0.15 M NaCl, 25 °C.

**Figure 3:**
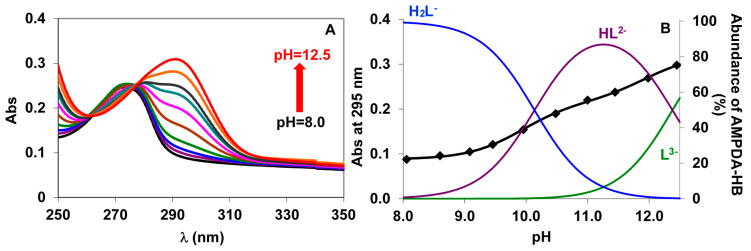
(**A**) Absorption spectra and (**B**) species distribution and the absorbance values at 295 nm of the AMPDA-HB ligand as a function of pH ([AMPDA-HB] = 1.0 × 10^−4^ M, l = 1.0 cm, 0.15 M NaCl, 25 °C).

The protonation sequence of AMPTA was determined through ^1^H-NMR spectroscopy by following the chemical shift of the non-labile protons as a function of pH (Figure 2). The ^1^H-NMR titration curves displayed large changes in the chemical shifts as a function of pH, which could be assigned to the protonation/deprotonation of the specific donor atoms in the AMPTA ligand. Since the protonation/deprotonation was fast on the NMR time scale, the chemical shifts of the observed signals could be expressed as a weighted average of the shifts of the different species involved in the protonation processes according to Equation (2) [25]:

(2) $$\delta_{H\left(obs\right)}=\sum^{\;}x_{i}\delta_{H}^{H_{i}L}$$

where $\delta_{H\left(obs\right)}$ is the observed chemical shift of a given signal, while x_i_ and $\delta_{H}^{H_{i}L}$ are the molar fractions and the chemical shifts of the involved species, respectively. The protonation/deprotonation of AMPDA-HB was studied using spectrophotometry on the absorption band of the aromatic group of the ligand, following the absorbance values at 295 nm. The UV spectra and the absorbance values at 295 nm of AMPDA-HB are shown in Figure 3. The absorbance of the ligand can be expressed as a sum of the absorption of each protonated species according to Equation (3) [26]:

(3) $$A=\sum [H_{i}L]\varepsilon_{H}^{H_{i}L}l$$

where *A* is the absorbance at a given wavelength; [*H_i_L*] and $\varepsilon_{H}^{H_{i}L}$ are the concentration and the molar absorptivity of the species, respectively; and *l* is the path length of the cell. The observed chemical shifts ($\delta_{H\left(obs\right)}$) and absorbance values (*A*) were fitted to Equations (2) and (3), respectively (the molar fractions *x*_i_ and the concentration of the different protonated species were expressed using the protonation constants *K_i_^H^*). The fittings of the experimental data points are shown in Figure 2 and Figure 3. The obtained log*K_i_^H^* values are listed in Table 1. 

**Scheme 3:**
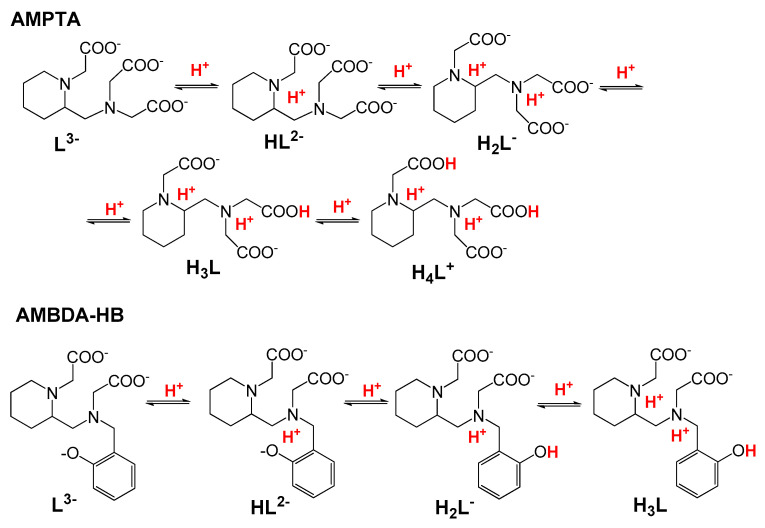
Protonation scheme of the AMPTA and AMPDA-HB ligands.

The ^1^H-NMR spectra of AMPTA at pH > 10 contained several broad multiplets and only a few of them were used to determine the protonation sequence of the AMPTA ligand (Figure 2). Starting from basic pH, the addition of one equivalent of acid to AMPTA resulted in a significant downfield shift of the signals of *e*, *g* and *f* protons, indicating that the first protonation took place at the nitrogen atoms of the piperidine group (the protonation occurred partially at the N atom of the piperidine and the iminodiacetic acid (IMDA) group). In the pH range of 4–8, the signals of the *g*, *h* and *f* protons were mainly affected by the second protonation process, which occurred at the N atom of the IMDA group. However, the signal of the *e* proton of the piperidine group was also shifted slightly to higher frequencies (downfield), which could be explained by the fact that, parallel with the protonation of the IMDA nitrogen, the first proton was transferred to the piperidine nitrogen due to the electrostatic repulsion between the protonated nitrogen atoms. At pH < 4, the downfield shift of the signals of the *g*, *h* and *f* protons confirmed that the log*K*_3_*^H^* and log*K*_4_*^H^* were related to the protonation of the IMDA carboxylates and the piperidine nitrogen, respectively (Scheme 3). The values of log*K*_1_*^H^* and log*K*_2_*^H^* obtained from the ^1^H NMR study agreed well with the related protonation constants of AMPTA, as determined using pH potentiometry (Table 1). 

The spectrophotometric study of AMPDA-HB over a range of pHs (Figure 3) revealed that the protonation of the ligand resulted in a significant decrease of the absorbance values at 295 nm. The protonation of L^3−^ and HL^2−^ resulted in the decrease of the absorbance values by 0.1 and 0.14 at 295 nm. According to the ΔAbs values, it could be assumed that the first protonation took place at the nitrogen that was functionalized with the hydroxybenzyl group (the protonation occurred partially at the N atom and the phenolate -O¯ group due to the H-bond formation), whereas the second protonation process of AMPDA-HB took place on the phenolate -O¯ group in the pH range 8–11. Comparison of the protonation constant of phenol (log*K*_H_ = 10.0, 0.1 M NaClO_4_, 25 °C) [27] with the log*K*_2_*^H^* value of AMPDA-HB also confirmed that the second protonation of AMPDA-HB ligand took place at the phenolate -O^−^. Finally, the further protonations of AMPDA-HB occurred at the non-protonated piperidine nitrogen atom and the carboxylate pendant arms (Scheme 3). The protonation constants of the AMPDA-HB ligand that were obtained by the spectrophotometric studies agreed well with the corresponding log*K_i_^H^* values that were determined using pH potentiometry (Table 1).

A comparison of the protonation constants of AMPTA and AMPDA-HB revealed that the log*K*_1_*^H^* value of AMPDA-HB was higher by about 0.8 log*K* units, which could be explained by the H-bond formation between the basic phenolate -O^¯^ and the protonated nitrogen atom. For comparison, the protonation constants of EDTA and CDTA were also determined (Table 1 and Supplementary Materials). Interestingly, the log*K*_1_*^H^* values of AMPTA and AMPDA-HB ligands were 2 log*K* units higher than those of the CDTA and EDTA ligands, which could be explained by the formation of Na(CDTA) and Na(EDTA) complexes, which resulted in the lower first protonation constants of these hexadentate ligands (Na(CDTA): log*K*_NaL_ = 4.66; Na(EDTA): log*K*_NaL_ = 1.43) [28,29]. The log*β*_4_*^H^* values, presented in Table 1, indicated that the total basicity of AMPDA-HB was about 8 orders of magnitude higher than that of AMPTA due to the presence of the very basic phenol group. However, the log*β*_4_*^H^* value of pentadentate AMPTA was comparable with that of hexadentate CDTA and EDTA ligands and significantly higher than the other pentadentate DPAMeA and DPAPhA ligands.

#### 2.2.2. Thermodynamic Properties of Mn(II) Complexes 

The stability and protonation constants of the Mn(II) complexes that were formed with AMPTA, AMPDA-HB, EDTA and CDTA are defined by Equations (4) and (5):

(4) $$K_{ML}=\frac{\left[ML\right]}{\left[M][L\right]}$$

(5) $$K_{MH_{i}L}=\frac{\left[MH_{i}L\right]}{\left[MH_{i-1}L][H^{+}\right]}\cdot \cdot \cdot \cdot \cdot \cdot i=1,2$$

The stability and protonation constants of the Mn(II) complexes were calculated from the titration curves obtained at 1:1 metal-to-ligand concentration ratios. The best-fitting curve was obtained by using the model that included the formation of *ML*, *MHL* and *MH*_2_*L* species in equilibrium. The stability and protonation constants of the Mn(AMPTA), Mn(AMPDA-HB), Mn(EDTA) and Mn(CDTA) complexes obtained using pH potentiometric titration are presented in Table 2 and compared to those of Mn(DPAMeA) and Mn(DPAPhA) [15].

The stability constant of Mn(AMPTA) was about 2.5 log*K* units smaller than that of Mn(AMPDA-HB) due to the lower total basicity of the AMPTA ligand (log*β*_4_*^H^*, Table 1). In contrast, the log*K*_MnL_ values of Mn(AMPTA) and Mn(AMPDA-HB) were higher by 1–1.5 and 3–4 log*K* units than those of the Mn(II) complexes that were formed with the pentadentate DPAMeA and DPAPhA ligands, respectively. The higher stability constants of the Mn(AMPTA) and Mn(AMPDA-HB) complexes could be explained by the higher basicity of the nitrogen donor atoms in the ligand backbone. Interestingly, the stability constant of Mn(AMPDA-HB) was similar to that of the Mn(II) complexes of hexadentate EDTA and CDTA ligands due to the presence of the basic phenolate group in the AMPDA-HB ligand. To compare the conditional stabilities, the pMn values of the complexes were calculated for identical conditions, i.e., [Mn^2+^]_tot_ = 1 μM, [L]_tot_ = 10 μM, pH = 7.4. The pMn value of Mn(AMPTA) was higher by about 0.5 log*K* units than that of the Mn(II) complexes with the pentadentate AMPDA-HB, DPAMeA and DPAPhA ligands; this could be explained by the higher total basicity of the AMPTA ligand than those of DPAMeA and DPAPhA, whereas the lower pMn value of Mn(AMPDA-HB) was caused by the presence of the protonated Mn(HL) species in the pH range of 4–6. The protonation of Mn(AMPDA-HB) occurred on the phenolic -O^−^ group, as confirmed via spectrophotometric studies on the Mn^2+^-AMPDA-HB system (Figure S9). The pMn value of Mn(AMPTA) was significantly smaller than that of Mn(II) complexes that were formed with hexadentate EDTA and CDTA ligands due to the lower denticity of the AMPTA ligand (one carboxylate group less than EDTA and CDTA).

### 2.3. ^1^H and ^17^O NMR Relaxometric Studies

**Figure 4:**
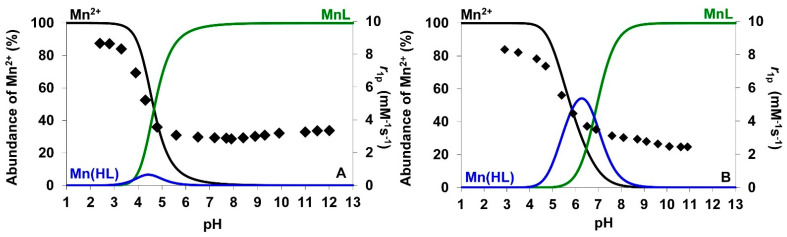
(**A**) Species distribution and relaxivity values (◆) as a function of pH of the Mn^2+^-AMPTA system; (**B**) species distribution and relaxivity values (◆) as a function of pH of the Mn^2+^-AMPDA-HB system; ([Mn^2+^] = [L] = 1.0 mM, 20 MHz, 0.15 M NaCl, 25 °C).

The proton relaxivity (*r*_1_) measures the enhancement of the relaxation rate of water proton nuclei in the presence of 1 mM paramagnetic agent solution. The relaxivity values for [Mn(AMPTA)]^−^ and [Mn(AMPDA-HB)]^−^ that were recorded at pH 7.4 (298 K, 20 MHz) were 3.3 and 3.4 mM^−1^ s^−1^, respectively. These values are in line with those reported for monohydrated Mn(II) complexes, such as [Mn(EDTA)]^2−^ (3.3 mM^−1^ s^−1^) [9] and [Mn(DPAA)]^−^ (3.5 mM^−1^ s^−1^, Table 3). *r*_1_ values as a function of pH and the species distribution obtained with the equilibrium constants (Table 1 and Table 2) characterizing the Mn^2+^-AMPTA and Mn^2+^-AMPDA-HB systems are shown in Figure 4. The relaxivity of [Mn(AMPTA)]^-^ remained constant in a broad pH range (ca. 5–11) and increased below pH 5 due to the dissociation of the Mn(II) complex (Figure 4). A different pH dependence of the *r*_1_ values was reported for [Mn(AMPDA-HB)]^-^, which showed a slight decrease in *r*_1_ from pH 6 to 11 (from 3.7 to 2.4 mM^−1^ s^−1^) and an increase in *r*_1_ below pH 6 due to the protonation of the Mn(II) complex, followed by Mn^2+^ dissociation at lower pH. 

**Figure 5:**
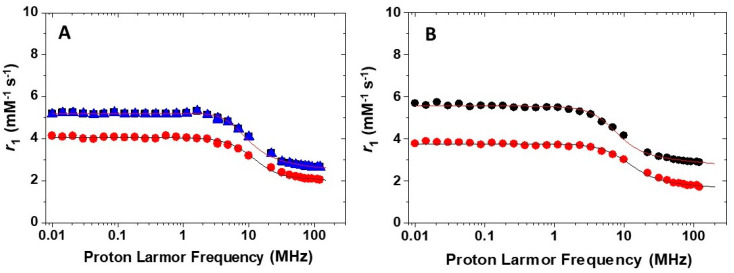
^1^H NMRD profiles recorded at 298 (●) and 310 K (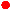) for (**A**) [Mn(AMPTA)]^−^ and (**B**) [Mn(AMPDA-HB)]^−^. The blue triangles represent the ^1^H NMRD profile at 298 K of enantiomerically pure [Mn((*R*)-AMPTA)]^−^. The lines represent the fit of the data, as explained in the text.

^1^H NMRD (proton nuclear magnetic relaxation dispersion) profiles of aqueous solutions of [Mn(AMPTA)]^−^ and [Mn(AMPDA-HB)]^−^ (Figure 5) were recorded at 298 and 310 K in the range of magnetic field strengths of 2.3 × 10^−4^ to 3.0 T, which corresponds to proton Larmor frequencies of 0.01–127 MHz. The NMRD profiles reproduced the typical shape of low molecular weight complexes in the fast water exchange regime, whose relaxivity was largely dominated by rotational dynamics. Moreover, the *r*_1_ values at 310 K were lower than those measured at 298 K over the entire range of investigated proton Larmor frequencies, indicating that *r*_1_ was not limited by the water exchange rate but rather by the fast rotational motion of the complex.

The NMRD profile at 298 K of the enantiomerically pure [Mn((*R*)-AMPTA)]^−^ was also acquired (Figure 5), which was exactly superimposable onto the NMRD profile of the racemic Mn(II) complex. Therefore, we can conclude that the two enantiomers did not exhibit substantial differences in terms of structural and relaxometric properties, as expected.

As proposed by Geraldes and Peters [30], the hydration number *q* can be determined by the correlation between the relaxivity measured at 0.01 MHz (and 298 K) and the molecular weight of the complex. For both [Mn(AMPTA)]^−^ and [Mn(AMPDA-HB)]^−^, the calculation gave *q* = 0.9, confirming the formation of monohydrated complexes in solution. This result appeared to contrast with the bis-hydration that was claimed for Mn complexes with pentadentate PAADA and DPAMeA ligands (Figure 1 and Table 3). We can assume that the heterocyclic piperidine ring hindered the coordination of a second water molecule more markedly than a flat pyridine ring. 

**Figure 6:**
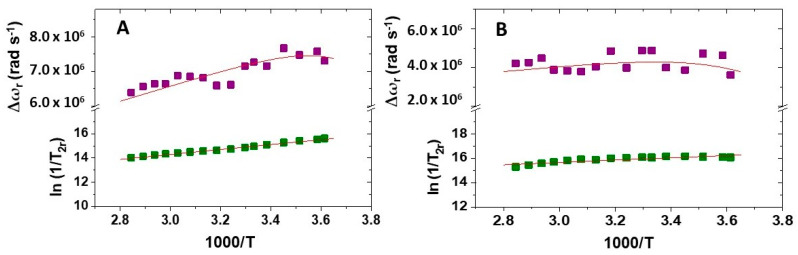
Reduced transverse ^17^O NMR relaxation rates and chemical shifts versus reciprocal temperature measured for (**A**) [Mn(AMPTA)]^−^ and (**B**) [Mn(AMPDA-HB)]^−^ at 11.74 T. The lines represent the fit of the data, as explained in the text.

In addition, the reduced transverse ^17^O NMR relaxation rates (1/*T*_2r_) and chemical shifts (Δω_r_) of [Mn(AMPTA)]^−^ and [Mn(AMPDA-HB)]^−^ were measured as a function of temperature to gain information on the exchange rate of the coordinated water molecule. The two complexes presented a slight increase in the 1/*T*_2r_ value with decreasing temperature over the full temperature range, which is typical of systems under the fast exchange regime (Figure 6).

The simultaneous fit of the ^1^H NMRD and ^17^O NMR data (the equations used in the fitting are summarized in the Supplementary Materials) afforded the structural and dynamic molecular parameters shown in Table 3, which were compared to the parameters reported previously for related Mn(II) complexes. Some of the parameters were fixed during the fit: the distance of closest approach for the outer-sphere contribution *a*_MnH_ at 3.6 Å; the distance between the Mn(II) ion and the proton nuclei of the coordinated water molecules (*r*_MnH_) at 2.83 Å; the diffusion coefficient *D*^298^ (2.24 × 10^−5^ cm^2^ s^−1^) and its activation energy *E*_D_ (20 kJ mol^−1^) were fixed to common values, while the number of water molecules in the inner coordination sphere of Mn(II) was fixed to *q* = 1 (Table 3).

The water exchange rate that was obtained for [Mn(AMPTA)]^−^ and [Mn(AMPDA-HB)]^−^ (^298^*t*_M_ = 1/*k*_ex_ = 1.8 ± 0.3 and 2.5 ± 0.3 ns, respectively) was fast, as already noted for the majority of Mn(II) complexes. The rotational correlation times (^298^*τ*_R_) and the ^17^O hyperfine coupling constants (A_O_/ħ) for both complexes fell within the range that is typically observed for small Mn(II) complexes. Finally, the parameters that are related to the electron spin relaxation of the metal ion (the electronic correlation time for the modulation of the zero-field-splitting interaction *τ*_V_ and the mean square zero-field-splitting energy Δ^2^) were also similar to those obtained for other Mn(II) complexes (Table 3).

### 2.4. Computational Modelling

**Figure 7:**
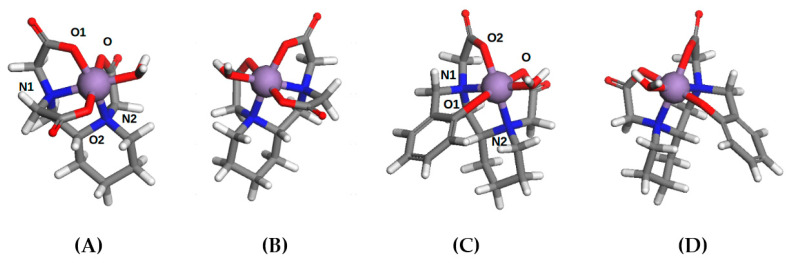
Structures optimized using DFT calculations: (**A**) [Mn((*S*)-AMPTA)·(H_2_O)]^−^; (**B**) [Mn((*R*)-AMPTA)·(H_2_O)]^−^; (**C**) [Mn((*S*)-AMPDA-HB)·(H_2_O)]^−^; (**D**) [Mn((*R*)-AMPDA-HB)·(H_2_O)]^−^.

The geometries of [Mn((*R*/*S*)-AMPTA)·(H_2_O)]^−^ and [Mn((*R*/*S*)-AMPDA-HB·(H_2_O)]^−^ were optimized at the DFT level, where an octahedral coordination environment was found around the Mn^2+^ ion, as shown in Figure 7. The calculated bond distances from Mn^2+^ are reported in the Supplementary Materials (Tables S1 and S2): in particular, the coordinated water molecule was found at 2.093 Å in the AMPTA- and 2.103 Å in the AMPDA-HB complex.

**Figure 8:**
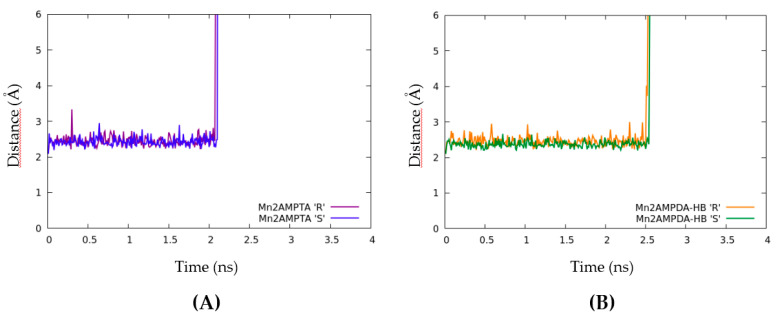
Time evolution of the distance between Mn^2+^ ion and the coordinated water molecule for (**A**) [Mn((*R*/*S*)-AMPTA)·(H_2_O)]^−^ and (**B**) [Mn((*R*/*S*)-AMPDA-HB·(H_2_O)]^−^.

Then, a series of MD simulations were performed on all the systems, in particular, to monitor the distance between the Mn(II) ion and the oxygen of the coordinated water molecule to estimate the residence time. The time evolution of the Mn–O_water_ distances for the two complexes are reported in Figure 8: from the computed dynamics, the residence time was 2.07 ns for [Mn((*R*/*S*)-AMPTA)·(H_2_O)]^−^ and 2.50 ns for [Mn((*R*/*S*)-AMPDA-HB·(H_2_O)]^−^. The former result was in excellent agreement with the experimental τ_m_ discussed above (2.1 ns), and the model predicted that the phenolate group would strengthen the coordination bond of water to the metal, resulting in a longer residence time.

**Figure 9:**
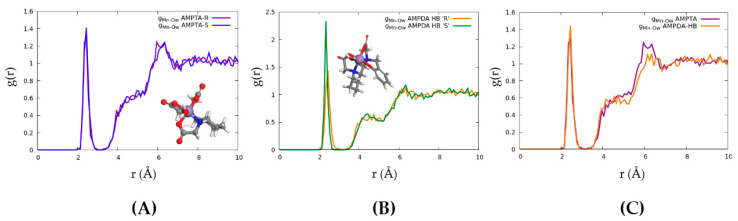
Radial distribution function of water oxygen relative to Mn(II). (**A**): [Mn((*R*/*S*)-AMPTA)·(H_2_O)]^−^; (**B**) [Mn((*R*/*S*)-AMPDA-HB·(H_2_O)]^−^; (**C**) comparison of g(Ow–Mn) for the *R*-enantiomer of both complexes.

Some insights about the solvent microscopic structure around the complexes were provided by the radial distribution function (*g*) of water oxygen (O_w_) relative to Mn(II), averaged over the MD configurations. In Figure 9, we report a comparison of *g*(O_w_–Mn) for the two complexes: at large distances, *g* tended to unity, corresponding to the bulk value; for both complexes, a sharp peak due to the water molecule coordinated to Mn(II) is present at 2.1–2.5 Å, then a depletion region followed up to 4 Å, corresponding to the solute molecule excluded volume. Finally, a rather broad peak was detected around 6 Å, showing a weakly structured solvation shell around the complexes; this last peak was less pronounced in [Mn(AMPDA-HB)·(H_2_O)]^−^ due to the greater steric hindrance of the phenyl group.

## 3. Materials and Methods

All chemicals were purchased from commercial sources and were used without purification. Water was purified (18 MΩ cm) using a standard Milli-Q system (Millipore, Bedford, MA, USA). “PE” (petroleum ether) refers to petroleum ether with a boiling point in the range of 40–60 °C. NMR spectra (including ^1^H-decoupled ^13^C NMR) were recorded on a Bruker Avance III spectrometer (Bruker, Milano, Italy) operating at 11.74 T and 298 K, corresponding to a protonic resonance frequency of 499.8 MHz. ^1^H and ^13^C NMR chemical shifts are reported relative to TMS and are referenced using the residual proton solvent resonances. Samples were prepared in 5 mm NMR tubes by dissolving the compounds in appropriate deuterated solvents. Splitting patterns are described as singlet (s), broad singlet (bs), doublet (d), double doublet (dd), triplet (t) or multiplet (m). ESI mass spectra were recorded on a Waters SQD 3100 (Waters Corporation, Milford, MA, USA). Analytical HPLC-MS was carried out on a Waters modular system equipped with Waters 1525 binary pump, Waters 2487 UV/Vis and Waters SQD 3100 (ESCI ionization mode) detectors using an XBridge^TM^ Phenyl 3.5 μm 4.6 mm × 150 mm column (Waters). Semi-preparative HPLC purifications were performed with a XBridge^TM^ Prep Phenyl 5 μm OBD^TM^ 19 mm × 100 mm column (Waters). The HPLC methods are indicated for each procedure (Table 4).

### 3.1. Synthesis

#### 3.1.1. Synthesis of 2-(Methoxymethoxy)benzaldehyde (1)

Salicylaldehyde (1.00 mL, 9.58 mmol) was added to a 2.1 M solution of MOM-Cl in toluene (9 mL, 19.16 mmol), followed by DIPEA (2.08 mL, 11.97 mmol), and the mixture was stirred at room temperature for 3 h. Then, 1 M NH_4_Cl (3 mL) was added and stirring was continued for a further 10 min; the reaction mixture was transferred into a separatory funnel, where the organic phase was separated and the water phase was extracted with EtOAc (5 mL). The combined organic phases were washed with saturated NaHCO_3_ (5 mL) and H_2_O (5 mL), dried over anhydrous MgSO_4_, filtered and evaporated under vacuum. Pale yellow oil: 1.3 g (81%).

^1^H-NMR (500 MHz, 25 °C, CDCl_3_), δ (ppm): 10.51 (CHO), 7.84 (dd, ^3^*J*_HH_ = 7.7 Hz, ^4^*J*_HH_ = 1.7 Hz, CH^Ar^), 7.52 (dt, ^3^*J*_HH_ = 8.5 Hz, ^4^*J*_HH_ = 1.7 Hz, CH^Ar^), 7.22 (d, ^3^*J*_HH_ = 8.5 Hz, 1H, CH^Ar^), 7.08 (t, ^3^*J*_HH_ = 7.7 Hz, 1H, CH^Ar^), 5.31 (s, 2H, OCH_2_O), 3.53 (s, 3H, CH_3_). ^13^C NMR (125 MHz, 25 °C, CDCl_3_), δ (ppm): 189.7 (CHO), 159.7 (COCH_2_), 135.9 (CH^Ar^), 128.4 (CH^Ar^), 125.5 (CCHO), 121.9 (CH^Ar^), 115.1 (CH^Ar^), 94.6 (CH_2_), 56.5 (CH_3_). 

#### 3.1.2. Synthesis of 2-(N-(2-(Methoxymethoxy)benzyl)aminomethyl)piperidine (2) 

2-Aminomethylpiperidine (1 mL, 8.24 mmol) was dissolved in dry MeOH (20 mL), AcOH (2 drops) was added, followed by intermediate **1** (1.37 g, 8.24 mmol), which was also dissolved in dry MeOH (5 mL) and added dropwise to the previous solution. The resulting yellow mixture was stirred at room temperature for 1 h, then NaBH_4_ (1.50 g, 39.65 mmol) was added portion-wise at 0 °C and the mixture was stirred for a further 2 h while turning colorless. H_2_O (1 mL) was added and the suspension was agitated for 30 min before removing the solvents under reduced pressure. The residue was suspended in EtOH (20 mL), stirred for 30 min and filtered. The filtrate was dried under vacuum, leading to the crude product as a yellow oil (1.57 g, 72%).

^1^H-NMR (500 MHz, 25 °C, CDCl_3_), δ (ppm): 7.26 (d, ^3^*J*_HH_ = 7.7 Hz, 1H, CH^Ar^), 7.21 (t, ^3^*J*_HH_ = 7.7 Hz, 1H, CH^Ar^), 7.08 (d, ^3^*J*_HH_ = 7.7 Hz, 1H, CH^Ar^), 6.97 (t, ^3^*J*_HH_ = 7.7 Hz, 1H, CH^Ar^), 5.22 (s, 2H, OCH_2_O), 3.80 (s, 2H, NCH_2_Ar), 3.49 (s, 3H, CH_3_), 3.06 (m, 1H, CHCHH’N), 2.6–2.5 (m, 3H, CHCHH’N + NCHH’CH_2_ + CHCH_2_N), 2.47 (m, 1H, NCHH’CH_2_), 1.95 (bs, 1H, NH), 1.79 (m, 1H, NCH_2_CHH’), 1.58 (m, 2H, CHCHH’CHH’), 1.5–1.3 (m, 2H, NCH_2_CHH’ + CHCHH’CH_2_), 1.09 (m, 1H, CHCH_2_CHH’). ^13^C NMR (125 MHz, 25 °C, CDCl_3_), δ (ppm): 149.7 (COCH_2_), 129.9 (CH^Ar^), 129.3 (CCH_2_), 128.2 (CH^Ar^), 121.6 (CH^Ar^), 113.9 (CH^Ar^), 94.4 (OCH_2_O), 56.5 (CH_3_), 56.1 (NCH), 55.1 (NCH_2_CH_2_), 49.3 (NCH_2_Ar), 46.7 (CHCH_2_N), 30.8 (CHCH_2_CH_2_), 26.6 (CHCH_2_CH_2_), 24.7 (NCH_2_CH_2_). ESI^+^ MS: *m/z* 265.1 [M + H^+^], calc. for [C_15_H_25_N_2_O_5_]^+^ = 265.19 g/mol.

#### 3.1.3. Synthesis of 2-(N-(2-(Methoxymethoxy)benzyl)aminomethyl)piperidine-N,N′-di-tert-butyl acetate (3)

Intermediate **2** (0.22 g, 0.83 mmol) was dissolved in CH_3_CN (10 mL). Na_2_CO_3_ (0.26 g, 2.49 mmol) and was added, followed by *tert*-butyl bromoacetate (0.49 mL, 3.32 mmol), and the mixture was stirred at room temperature overnight. The solvent was evaporated under reduced pressure and the residue was suspended in EtOAc (20 mL) and washed with H_2_O (2 × 10 mL) and brine (10 mL). The organic phase was dried over anhydrous MgSO_4_, filtered and evaporated under vacuum, and the product was obtained as a pale yellow oil (0.33 g, 82%).

^1^H-NMR (500 MHz, 25 °C, CDCl_3_), δ (ppm): 7.36 (dd, ^3^*J*_HH_ = 7.5 Hz, ^4^*J*_HH_ = 1.5 Hz, 1H, CH^Ar^), 7.18 (m, 1H, CH^Ar^), 7.07 (d, ^3^*J*_HH_ = 8.2 Hz, ^4^*J*_HH_ = 1.0 Hz, 1H, CH^Ar^), 6.95 (m, 1H, CH^Ar^), 5.18 (d, ^2^*J*_HH_ = 6.7 Hz, 1H, OCHH’O), 5.16 (d, ^2^*J*_HH_ = 6.7 Hz, 1H, OCHH’O), 3.83 (d, ^2^*J*_HH_ = 13.7 Hz, NCHH’Ar), 3.78 (d, ^2^*J*_HH_ = 13.7 Hz, NCHH’Ar), 3.47 (m, 5H, NCH_2_CO + OCH_3_), 3.24 (d, ^2^*J*_HH_ = 16.9 Hz, 1H, NCHH’CO), 3.19 (d, ^2^*J*_HH_ = 16.9 Hz, 1H, NCHH’CO), 2.95 (dd, ^2^*J*_HH_ = 13.1 Hz, ^3^J_HH_ = 5.1 Hz, 1H, CHCHH’N), 2.78 (m, 2H, NCH + NCHH’CH_2_), 2.64 (dd, ^2^*J*_HH_ = 13.1 Hz, ^3^*J*_HH_ = 5.8 Hz, 1H, CHCHH’N), 2.55 (m, 1H, NCHH’CH_2_), 1.83 (m, 1H, CHCH_2_CHH’), 1.65 (m, 1H, NCH_2_CHH’), 1.55 (m, 1H, CHCH_2_CH_2_), 1.45 (s, 9H, 3 × CH_3_^tBu^), 1.42 (s, 9H, 3 × CH_3_^tBu^), 1.28 (m, 2H, NCH_2_CHH’ + CHCH_2_CHH’). ^13^C NMR (125 MHz, 25 °C, CDCl_3_), δ (ppm): 170.9 (C=O), 155.7 (COCH_2_), 130.9 (CH^Ar^), 128.2 (CH^Ar^), 127.8 (CCH_2_), 121.5 (CH^Ar^), 114.1 (CH^Ar^), 94.5 (OCH_2_O), 80.6 (C^tBu^), 57.9 (CHCH_2_N), 57.4 (NCH), 56.1 (NCH_2_CO), 56.0 (OCH_3_), 55.3 (NCH_2_CO), 53.5 (NCH_2_CH_2_), 52.6 (NCH_2_Ar), 30.9 (CHCH_2_CH_2_), 28.1 (CH_3_^tBu^), 25.7 (CHCH_2_CH_2_), 23.7 (NCH_2_CH_2_). ESI^+^ MS: *m/z* 493.4 [M + H^+^] (calc. for [C_27_H_45_N_2_O_6_]^+^: 493.33 g/mol).

#### 3.1.4. Synthesis of AMPDA-HB 

Intermediate **3** (0.33 g, 0.67 mmol) was dissolved in TFA (3 mL) and the mixture was stirred at room temperature for 3 h. The solvent was evaporated under reduced pressure and the residue was dissolved again in TFA (1 mL) and precipitated in Et_2_O (10 mL). The suspension was centrifuged (4000 rpm, 15 min, 10 °C) and the precipitate was washed/centrifuged with Et_2_O (3 × 10 mL) and dried under vacuum. The product was purified using semi-preparative HPLC-MS and obtained after lyophilization as a white monotrifluoroacetate salt (0.16 g, 53%).

The characterization data (HPLC MS, ^1^H and ^13^C NMR) were in agreement with those previously reported [18].

#### 3.1.5. Synthesis of (R)-2-(Aminomethyl)piperidine (6)

Amide **5** (0.54 g, 4.22 mmol) was dissolved in dry THF (2 mL) and a 2 M solution of LiAlH_4_ in THF (6.33 mL, 12.66 mmol) was added at 0 °C. After stirring for 10 min, the mixture was heated to reflux for 3 h; then it was left to cool down to room temperature and the flask was placed in an ice bath. H_2_O (1 mL) was added dropwise slowly and the resulting suspension was stirred for a further 30 min and then filtered. After removal of the solvents from the filtrate under reduced pressure, the residue was suspended in EtOAc (20 mL) and filtered again, where the filtrate was dried over anhydrous MgSO_4_, filtered and evaporated under vacuum, leading to the intended compound **6** as a white solid (0.42 g, 87%).

The characterization data (ESI MS, ^1^H and ^13^C NMR) were consistent with those obtained from the commercial racemic analogous compound.

#### 3.1.6. Synthesis of (R)-2-(Aminomethyl)piperidine-N,N′,N′-tri-tert-butyl acetate (7)

Intermediate **6** (0.072 g, 0.63 mmol) was dissolved in MeCN (5 mL). K_2_CO_3_ (0.39 g, 2.84 mmol) was added, followed by *tert*-butyl bromoacetate (0.42 mL, 2.84 mmol), and the mixture was stirred at 50 °C overnight. The solvent was evaporated under reduced pressure and the residue was suspended in EtOAc (10 mL) and washed with H_2_O (2 × 5 mL) and brine (5 mL). The organic phase was dried over anhydrous MgSO_4_, filtered and evaporated under vacuum. The crude product was purified using flash chromatography (SiO_2_, PE/EtOAC 90:10→60:40, R_f_^80:20^ = 0.22), leading to the intended compound as a pale yellow oil (0.21 g, 74%).

The characterization data (ESI MS, ^1^H and ^13^C NMR) were consistent with those reported for the analogous racemic compound [18].

#### 3.1.7. Synthesis of (R)-2-AMPTA 

Intermediate **7** (0.29 g, 0.63 mmol) was dissolved in DCM (5 mL). TFA (5 mL) was added and the mixture was stirred at rt for 15 h. The solvents were evaporated under reduced pressure and the residue was dissolved again in TFA (1 mL) and precipitated in Et_2_O (10 mL). The suspension was centrifuged (4000 rpm, 15 min, 10 °C) and the precipitate was washed/centrifuged with Et_2_O (3 × 10 mL). The product was purified using semi-preparative HPLC-MS, and the product was obtained as a white solid (54 mg, 30%). 

The characterization data (ESI MS, ^1^H and ^13^C NMR) were consistent with those reported for the analogous racemic compound [18].

### 3.2. Equilibrium Measurements

The chemicals used for the experiments were of the highest analytical grade. The MnCl_2_ solutions were prepared from MnCl_2_·4H_2_O (*Sigma*, St. Louis, MI, USA; 99.9%). The concentration of the MnCl_2_ solution was determined via complexometric titration with standardized Na_2_H_2_EDTA and eriochrome black T as the indicator. The concentrations of the H_3_AMPTA, H_2_AMPDA-HB, H_4_CDTA (Sigma, 99.9 %) and H_4_EDTA (Sigma, 99.9%) were determined using pH potentiometric titration in the presence and absence of a large (40-fold) excess of CaCl_2_. The pH potentiometric titrations were made with standardized 0.2 M NaOH.

The stability and protonation constants of the Mn(II) complexes formed with AMPTA, AMPDA-HB, EDTA and CDTA ligands were determined using pH potentiometric studies. For the pH measurements and titrations, a Metrohm 888 Titrando automatic titration workstation combined electrode (Metrohm-6.0234.110, Metrohm, Herisau, Switzerland) was used. Equilibrium measurements were carried out at a constant ionic strength (0.15 M NaCl) in 6 mL samples at 25 °C. The solutions were stirred and N_2_ was bubbled through them. The titrations were made in the 1.7–12.0 pH range. KH-phthalate (pH = 4.008) and borax (pH = 9.180) buffers were used to calibrate the pH meter. For the calculation of [H^+^] from the measured pH values, the method proposed by Irving et al. [31] was used as follows: a 0.01 M HCl solution was titrated with standardized NaOH solution at 0.15 M NaCl ionic strength; the differences (A) between the measured (pHread) and calculated pH (−log[H^+^]) values were used to obtain the equilibrium H^+^ concentration from the pH values measured in the titration experiments (A = 0.03). The waiting time between the two pH measurements was 60 sec. For the equilibrium calculations, the stoichiometric water ionic product (pKw) was also needed to calculate [OH^−^] values under basic conditions. The V_NaOH_–pH_read_ data pairs of the HNO_3_-NaOH titration obtained in the pH range 10.5–12.0 were used to calculate the p*K*_w_ value (p*K*_w_ = 13.85).

The protonation of the AMPDA-HB and the formation of the Mn(AMPDA-HB) complex were followed by spectrophotometric studies of the AMPDA-HB ligand and Mn^2+^-AMPDA-HB system at the absorption band of the phenyl group in the wavelength range of 210–350 nm. The concentrations of Mn^2+^ and AMPDA-HB were 0.1 mM. The absorption spectra of the AMPDA-HB ligand and Mn^2+^-AMPDA-HB solutions were recorded in the pH range of 2.0–12.5. All spectrophotometric measurements were performed at 25 °C in 0.15 M NaCl solution. The pH was adjusted via the stepwise addition of concentrated NaOH or HCl solutions. The spectrophotometric measurements were made with the use of a PerkinElmer Lambda 365 UV-Vis spectrophotometer (PerkinElmer, Waltham, MA, USA) using 1.0 cm cells. The protonation and stability constants were calculated with the PSEQUAD program [32].

The protonation process of the H_3_AMPTA ligand was also followed by ^1^H NMR spectroscopy. For these experiments, a 0.01 M solution of the ligand in D_2_O was prepared in the presence of 0.15 M NaCl. The pH was adjusted via the stepwise addition of a solution of NaOD or DCl (both prepared in D_2_O). The pH values reported for the ligand were corrected for the deuterium effect by using the relationship pD = pH + 0.4 [33]. The calculations were performed using the computer program Micromath Scientist, version 2.0 (Salt Lake City, UT, USA).

### 3.3. ^1^H NMRD and ^17^O NMR Measurements

The Mn^2+^ complexes were prepared by mixing solutions of MnCl_2_ and the ligand (in ca. 5% molar excess) and adjusting the pH to 7.4 with HCl or NaOH. The exact concentration of the aqueous solutions for the ^1^H NMRD and ^17^O NMR measurements was determined by measuring the bulk magnetic susceptibility shifts of the t-BuOH ^1^H NMR signal at 11.7 T [34]. Proton relaxation measurements (1/T_1_) and the resulting 1/T_1_ NMRD profiles were measured on a Fast-Field Cycling (FFC) Stelar SmarTracer Relaxometer (Stelar s.r.l., Mede (PV), Italy) over a continuum of magnetic field strengths from 0.00024 to 0.25 T (corresponding to 0.01–10 MHz proton Larmor frequencies). The relaxometer operates under computer control with an absolute uncertainty in 1/T_1_ of ±1%. Precise control of the temperature was achieved during the measurements by means of a Stelar VTC-91 airflow heater equipped with a calibrated copper-constantan thermocouple (uncertainty of ± 0.1 °C). Furthermore, the real temperature inside the probe head was additionally monitored using a Fluke 52 k/j digital thermometer (Fluke, Zürich, Switzerland). Additional data in the 20–120 MHz frequency range were obtained with a High Field Relaxometer (Stelar) equipped with the HTS-110 3T Metrology Cryogen-free Superconducting Magnet. The data were collected using the standard inversion recovery sequence (20 experiments, 2 scans) with a typical 90° pulse width of 3.5 ms, and the reproducibility of the data was within ± 0.5%.

^17^O NMR spectra were recorded on a Bruker Avance III spectrometer (11.7 T) equipped with a 5 mm probe and a standard temperature control unit. Aqueous solutions of the complexes (ca. 6–10 mM) containing 2.0% of the ^17^O isotope (Cambridge Isotope) were used. The observed transverse relaxation rates were calculated from the signal width at half-height as a function of temperature in the 278–350 K range. 

### 3.4. Theoretical Models

DFT optimizations were performed with the NWCHEM program with the hybrid functional B3LYP [35]. For all atoms, the Ahlrichs’ cc-pVDZ basis set was used [36]. Solvent effects were included with the implicit conductor-like screening model (COSMO) with the water dielectric constant (ε = 78.4) [37]; dispersion energies were obtained with the semi-empirical approach proposed by Grimme [38].

MD simulations were performed with the LAMMPS software package [39] using the UFF force field [40]. The starting geometries of the metal complexes were obtained using DFT calculations, while the partial atomic charges were obtained with the ‘*Qeq*’ package implemented in Material Studio suite, using a convergence limit of 1.0 × 10^−6^ *e* (Material Studio 6.0; Accelrys Software Inc., San Diego, CA, 2011). We used the TIP3P model of Price et al. [41] to describe the solvent, fixing the atomic parameters of water molecules using the SHAKE procedure [42]. For each system, the solute molecule was immersed in a 25 Å × 25 Å × 25 Å cubic periodic box using the Packmol package to obtain a solution density of about 1.01 g/mL [43]. One Na^+^ counterion was added to neutralize the negative charge of the Mn complex. Van der Waals parameters for Na, optimized for the TIP3P model, were taken from the published work of Joung et al. [44]. During each MD, the first 5 ps of the NVE simulation was performed by allowing only water molecules to move, followed by a 4 ns NVT run at 298 K. The temperature was conserved using the Nosé–Hoover thermostat.

## 4. Conclusions

The Mn(II) complexes of two pentadentate ligands based on the (2-aminomethyl)-piperidine structure were investigated using potentiometry, spectrophotometry, ^1^H and ^17^O NMR relaxometry and DFT calculations. In particular, the ligand containing three acetate pendant arms (AMPTA) showed the lower overall basicity but higher stability of the Mn complex (pMn = 7.89), whereas the ligand with two acetate and one 2-hydroxybenzyl pendants (AMPDA-HB) showed the higher total basicity but lower stability of the metal complex due to the protonation of the phenol group at pH < 6 (pMn = 7.07). Both Mn complexes were mono-hydrated with relaxivities in the order of 3.3–3.4 mM^−1^ s^−1^ (at 20 MHz and 298 K). The water exchange rate for both complexes was very fast, with *τ*_M_ values around 2–2.5 ns; these values were confirmed using molecular dynamics calculations. Finally, DFT calculations allowed for confirming the octahedral coordination geometries for both Mn complexes. 

The present contribution expands the library of Mn-based chelates that are characterized in the context of MRI contrast agents as an alternative to the classical Gd(III) complexes, with the aim of obtaining improved and more reliable information on the correlation between solution structure and molecular relaxation parameters.

## Figures and Tables

**Table 1:** Protonation constants of AMPTA and AMPDA-HB compared to other ligands (0.15 M NaCl, 25 °C); standard deviations are shown in parentheses.

	AMPTA		AMPDA-HB		DPAMeA^15^	DPAPhA^15^	CDTA	EDTA
Meth.	pH Pot.	^1^H	pH Pot.	UV	pH Pot.	pH Pot.	pH Pot.	pH Pot.
log*K*_1_*^H^*	11.67 (1)	11.59 (3)	12.4 (1)	12.0 (1)	7.82	5.48	9.54 (1)	9.28 (1)
log*K*_2_*^H^*	5.47 (2)	5.59 (3)	10.14 (2)	9.92 (5)	3.71	4.51	5.97 (1)	6.04 (1)
log*K*_3_*^H^*	2.74 (2)	-	4.76 (3)	-	2.61	4.28	3.60 (1)	2.72 (1)
log*K*_4_*^H^*	1.62 (2)	-	1.91 (4)	-	-	2.70	2.52 (1)	1.99 (1)
log*K*_5_*^H^*	-	-	-	-	-	-	1.46 (1)	1.11 (6)
log*β*_4_*^H^*	21.49		29.19		14.14	16.97	21.63	20.03

**Table 2:** The stability and protonation constants of the Mn(II) complexes of AMPTA, AMPDA-HB, DPAMeA, DPAPhA, CDTA and EDTA ligands (0.15 M NaCl, 25 °C).

	AMPTA	AMPDA-HB	DPAMeA [16]	DPAPhA [16]	CDTA	EDTA
log*K*_MnL_	11.20 (1)	13.69 (4)	10.13	9.55	14.32 (1)	12.95 (1)
log*K*_MnHL_	3.77 (4)	6.80 (4)	2.57	4.84	2.90 (1)	2.65 (2)
log*K*_MnH2L_	-	5.60 (5)	-	2.51	1.89 (4)	2.51 (4)
pMn ^a^	7.89	7.07	7.28	7.27	13.11	12.00

**^a^** pMn = −log [Mn^2+^]_free_, [Mn^2+^]_tot_ = 1 μM, [L]_tot_ = 10 μM, pH = 7.4.

**Table 3:** Relaxometric parameters for Mn(AMPTA) and Mn(AMPDA-HB), which were obtained from the simultaneous analysis of ^17^O NMR and ^1^H NMRD data and compared to other relevant Mn(II) complexes.

	AMPTA	AMPDA-HB	DPAA [16]	DPAMeA [16]	PAADA [17]
*r_1_* at 25/37 °C, 20 MHz (mM^−1^ s^−1^)	3.3/2.6	3.4/2.7	3.5/2.7	5.3/4.2	4.0/3.3
$k_{R}^{\mathrm{298}}$ (10^7^ s^−1^)	56 ± 3	40 ± 5	12.6	30.6	90.0
Δ*H*^‡^ (kJ mol^−1^)	25.5 ± 0.5	28.3 ± 0.3	42.7	28.1	28.3
$\tau_{R}^{\mathrm{298}}$ (ps)	54.1 ± 0.9	60.6 ± 1.5	47.6	47.8	40.1
*E*_r_ (kJ mol^−1^)	20.2 ± 0.5	18.5 ± 1.1	22.8	25.3	22.6
$\tau_{v}^{\mathrm{298}}$ (ps)	27.8 ± 1.7	21.5 ± 1.4	19.4	39.2	12.0
Δ^2^ (10^19^ s^−2^)	7.3 ± 0.5	9.2 ± 0.6	5.5	2.4	12.8
*A*_O_/ħ (10^6^ rad s^−1^)	−28.6 ± 0.4	−33.0 ± 0.5	−31.5	−45.8	−38.6
*q* ^298^	1	1	1	2	2

**Table 4:** HPLC-UV analytical method for compound **1** (t_R_ = 11.1 min).

Solvent A = H_2_O (TFA 0.1%), Solvent B = ACN (TFA 0.1%), Flow = 1 mL/min.		
Time	%A	%B
0	70	30
2	70	30
16	0	100
19	0	100

## Data Availability

The data are available on request from the corresponding author.

## Supplementary Materials

The following are available online, Table S1: Calculated bond distances for Mn complexes with AMPTA, Table S2: Calculated bond distances for Mn complexes with AMPDA-HB, Figure S1: ^1^H-NMR spectrum in CDCl_3_ of protected salicylaldehyde **1**, Figure S2: ^13^C-NMR spectrum in CDCl_3_ of protected salicylaldehyde **1**, Figure S3: ^1^H-NMR spectrum in CDCl_3_ of intermediate **2**, Figure S4: ^13^C NMR spectrum in CDCl_3_ of intermediate **2**, Figure S5: ^1^H NMR spectrum in CDCl_3_ of intermediate **3**, Figure S6: ^13^C-NMR spectrum in CDCl_3_ of intermediate **3**, Figure S7: ESI^+^ MS, UV and specific ion HPLC chromatograms of AMPDA-HB prepared according to the new method, Figure S8: ESI^+^ MS, UV and specific ion HPLC chromatograms of (*R*)-AMPTA, Figure S9: Absorption spectra and absorbance values at 295 nm of the Mn^2+^-AMPDA-HB system as a function of pH, Figure S10: Species distribution and relaxivity values of the Mn^2+^-CDTA system as a function of pH.

## Sample Availability

Samples of the compounds are not available from the authors.
