# Supplementary material for: Semi-Rigid (Aminomethyl) Piperidine-Based Pentadentate Ligands for Mn(II) Complexation
Source: Molecules. 2021 Oct 2;26(19):5993. doi: 10.3390/molecules26195993 (PMC8513077; doi:10.3390/molecules26195993)
Supplement: Supplementary file 1 [file molecules-26-05993-s001.zip › molecules-1393685-supplementary.pdf]

# Supporting Information

# Semi-rigid (aminomethyl)piperidine-based pentadentate ligands for Mn(II) complexation

Jonathan Martinelli,[a] Edoardo Callegari,[a] Zsolt Baranyai,[b] Alberto Fraccarollo,[a] Maurizio Cossi,[a] Lorenzo Tei[a]*

[a] Dipartimento di Scienze e Innovazione Tecnologica, Università del Piemonte Orientale, Viale Michel 11, 15121 Alessandria, Italy;

[b] Bracco Research Center, Bracco Imaging SpA, Via Ribes 5, 10010 Colleretto Giacosa (TO), Italy;

## Table of contents

1. Fitting of $^{1}$H NMRD profiles and $^{17}$O NMR relaxivity data ........ 2

2. Computational modelling ........ 3

3. NMR spectra ........ 4

4. HPLC-MS ........ 7

5. Equilibrium studies of the Mn(II) complexes ........ 9

## 1. Fitting of ¹H NMRD profiles and ¹⁷O NMR relaxivity data

From the two contributions (inner sphere ($r_{1,IS}$) and outer-sphere ($r_{1,OS}$)) to the longitudinal proton relaxation rate ($r_1$) (eq. 1), the former one is considered for the fitting of $^1$H NMRD profiles (1).

$$r_1 = r_{1,IS} + r_{1,OS} \quad (1)$$

According to the Solomon–Bloembergen theory, the $r_{1,IS}$ is dominated by the dipolar interaction (eqs. 2 and 3), where $q$ is the number of water molecules directly coordinated to the Mn-ion, $T_{1M}$ is their longitudinal relaxation time, $\tau_M$ is the water-exchange correlation time (2-4).

$$r_{1,IS} = \frac{q}{55556(T_{1M}+\tau_M)} \quad (2)$$

$$\frac{1}{T_{1M}} = \frac{2}{15}\left(\frac{\mu_0}{4\pi}\right)^2 \frac{\hbar^2 \gamma_S^2 \gamma_I^2}{r_{MnH}^6} S(S+1)\left(\frac{3\tau_{d1}}{1+\omega_I^2\tau_{d2}^2}\right) \quad (3)$$

Here, $r_{MnH}$ is the distance between the proton of a bound $H_2O$ molecule and Mn-ion (fixed at 3.0 Å), $\mu_0/4\pi$) is the magnetic permeability in vacuum, $S$ is the electron spin (7/2), $\gamma_I$ and $\gamma_S$ are the gyromagnetic ratios, and $\omega_I$ and $\omega_S$ are the Larmor frequencies of the proton and electron, resp. $T_{ie}$ ($i$ = 1,2) represents electronic relaxation time that contributes to $\tau_{di}^{-1} = \tau_M^{-1} + \tau_R^{-1} + T_{ie}^{-1}$, expressed by eqs. 4 and 5 due to zero-field splitting (ZFS) (5).

$$\frac{1}{T_{1e}} = \frac{1}{25}\Delta^2\tau_V[4S(S+1)-3]\left(\frac{1}{1+\omega_S^2\tau_V^2} + \frac{1}{1+4\omega_S^2\tau_V^2}\right) \quad (4)$$

$$\frac{1}{T_{2e}} = \Delta^2\tau_V\left(\frac{5.26}{1+0.372\omega_S^2\tau_V^2} + \frac{7.18}{1+1.24\omega_S^2\tau_V^2}\right) \quad (5)$$

Here, $\Delta^2$ represents the mean-squared fluctuation of the ZFS, $\tau_V$ is the correlation time for the instantaneous distortion of the coordination polyhedron of Gd-ion. The outer-sphere contribution to the relaxivity ($r_{1,OS}$) is described by eqs. 6 and 7, where $N_A$ is the Avogadro's number, $a_{GdH}$ the distance of diffusing water molecule in the closest approach to Gd-ion (fixed at 4.0 Å), $D_{GdH}$ is diffusion coefficient and $J_{os}(\omega_S, Tje)$ ($j$ = 1, 2) are spin density functions (6).

$$r_{1,IS} = \left(\frac{32\pi}{405}\right)\left(\frac{\mu_0}{4\pi}\right)^2 \gamma_I^2\gamma_S^2\hbar^2 S(S+1)\frac{N_A}{a_{MnH}D_{MnH}}[3J_{OS}(\omega_I, T_{1e}) + 7J_{OS}(\omega_S, T_{1e})] \quad (6)$$

$$J_{OS}(\omega_I, T_{je}) = Re\left\{\frac{1+\frac{1}{4}\left[i\omega\tau_{MnH}+\left(\frac{\tau_{MnH}}{T_{je}}\right)\right]^{\frac{1}{2}}}{1+\left[i\omega\tau_{MnH}+\left(\frac{\tau_{MnH}}{T_{je}}\right)^{\frac{1}{4}}\right]+\frac{4}{9}\left[i\omega\tau_{MnH}+\left(\frac{\tau_{MnH}}{T_{je}}\right)\right]+\frac{1}{9}\left[i\omega\tau_{MnH}+\left(\frac{\tau_{MnH}}{T_{je}}\right)^{\frac{1}{2}}\right]}\right\} \quad (7)$$

All correlation times $\tau$ (X = R, M, V) exhibit an exponential dependence (eq. 8), where $E_X$ are activation energies (fixed at $E_V$ = 1 kJ mol$^{-1}$, $E_R$ = 1 kJ mol$^{-1}$) and R is the gas constant, leading to a temperature dependent diffusion coefficient $D$ (fixed at 2.24×10$^{-5}$ cm$^2$ s$^{-1}$ for 298.15K).

$$\tau_X^T = \tau_X^{298} exp\left[\frac{E_X}{R}\left(\frac{1}{T} - \frac{1}{298.15}\right)\right] \quad (8)$$

$$D_{MnH}^T = D_{MnH}^{298} exp\left[\frac{E_D}{R}\left(\frac{1}{298.15} - \frac{1}{T}\right)\right] \quad (9)$$

Mn-induced water $^{17}O$ longitudinal and transverse relaxation rates ($1/T_{1r}$ and$1/T_{2r}$) and angular frequencies ($\Delta_{\omega r}$) were calculated using eqs. 10 and 11 (7-8).

$$\frac{1}{T_{1r}} = \frac{1}{T_{1M}+\tau_M} \quad (10)$$

$$\frac{1}{T_{2r}} = \frac{1}{\tau_M}\frac{1}{T_{1M}+\tau_M} \quad (11)$$

## 2. Computational modeling

**Table S1:** Calculated bond distances for Mn complexes with AMPTA.

| **Mn((*S*)-AMPTA)** | Distance (Å) | **Mn((*R*)-AMPTA)** |
|---|---|---|
| 2.093 | Mn-$O_{water}$ | 2.093 |
| 2.138 | Mn-N2 | 2.137 |
| 2.074 | Mn-N1 | 2.075 |
| 2.020 | Mn-O | 2.021 |
| 2.008 | Mn-O1 | 2.008 |
| 2.054 | Mn-O2 | 2.054 |

**Table S2:** Calculated bond distances for Mn complexes with AMPDA-HB.

| **Mn((*S*)-AMPDA-HB)** | Distance (Å) | **Mn((*R*)-AMPDA-HB)** |
|---|---|---|
| 2.103 | Mn-$O_{water}$ | 2.103 |
| 2.145 | Mn-N2 | 2.145 |
| 2.078 | Mn-N1 | 2.078 |
| 2.042 | Mn-O | 2.042 |
| 2.000 | Mn-O1 | 2.000 |
| 2.067 | Mn-O2 | 2.067 |

## 3. NMR Spectra

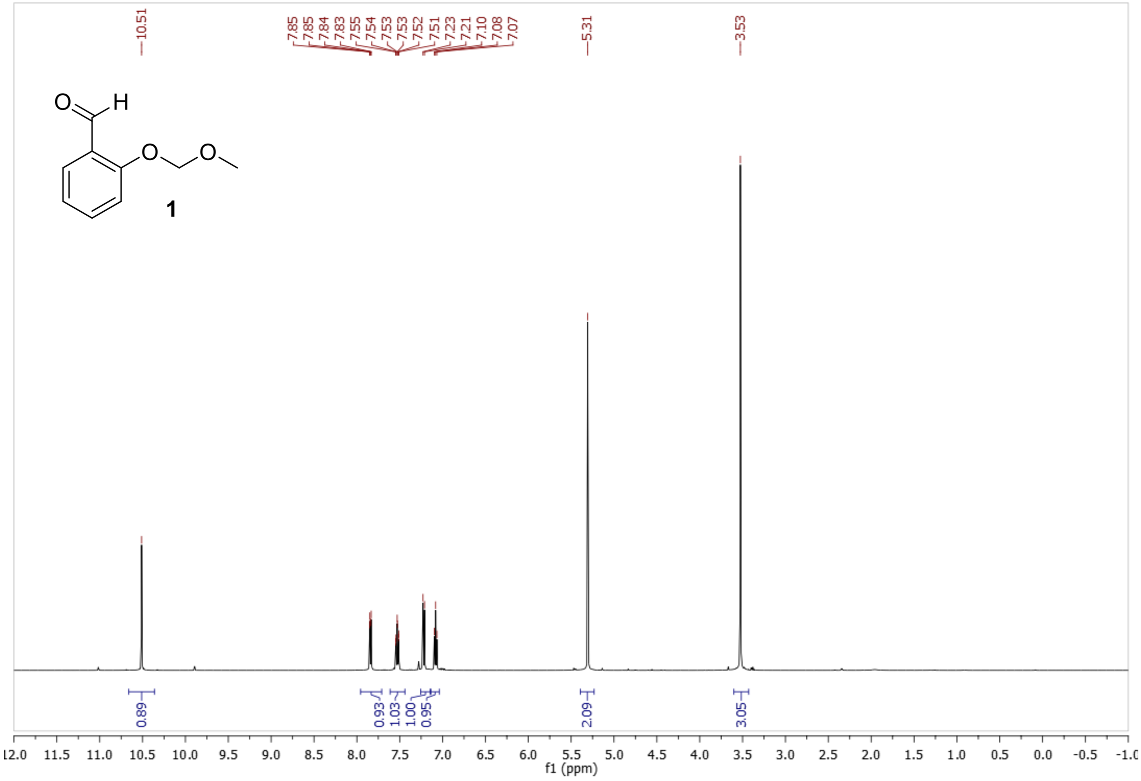


**Figure S1**. $^{1}$H NMR spectrum in $CDCl_3$ of protected salicylaldehyde **1**.

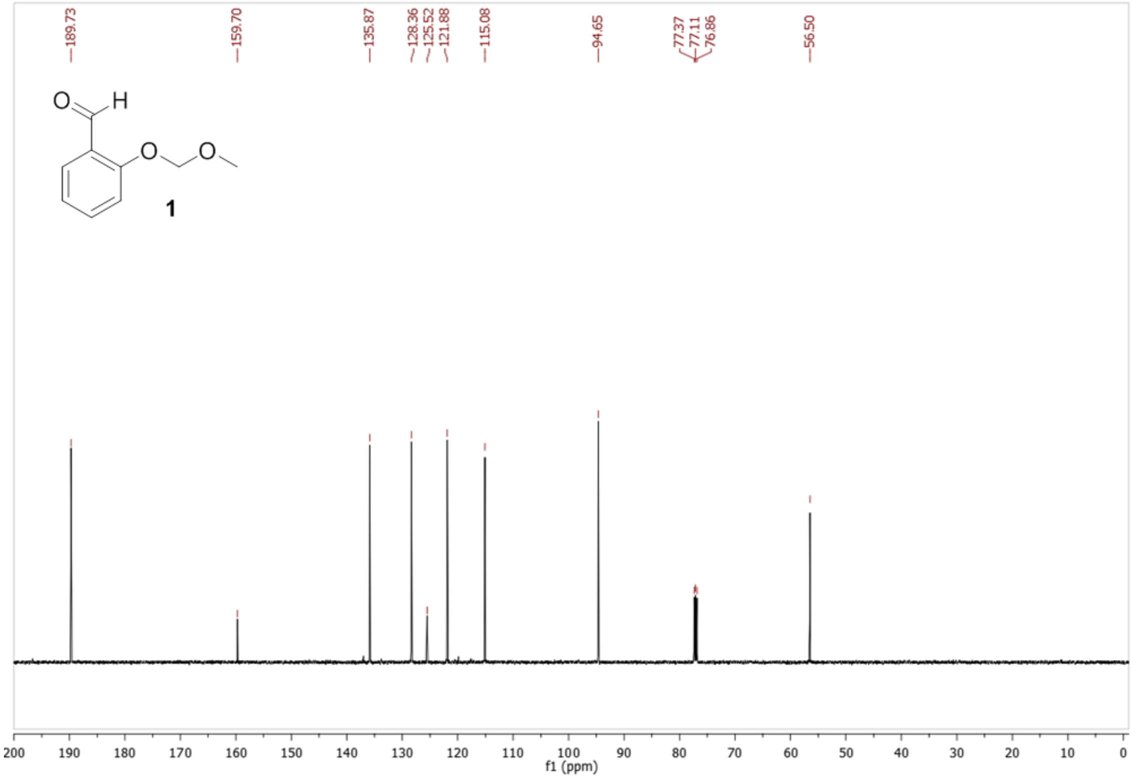


**Figure S2**. $^{13}$C NMR spectrum in $CDCl_3$ of protected salicylaldehyde **1**.

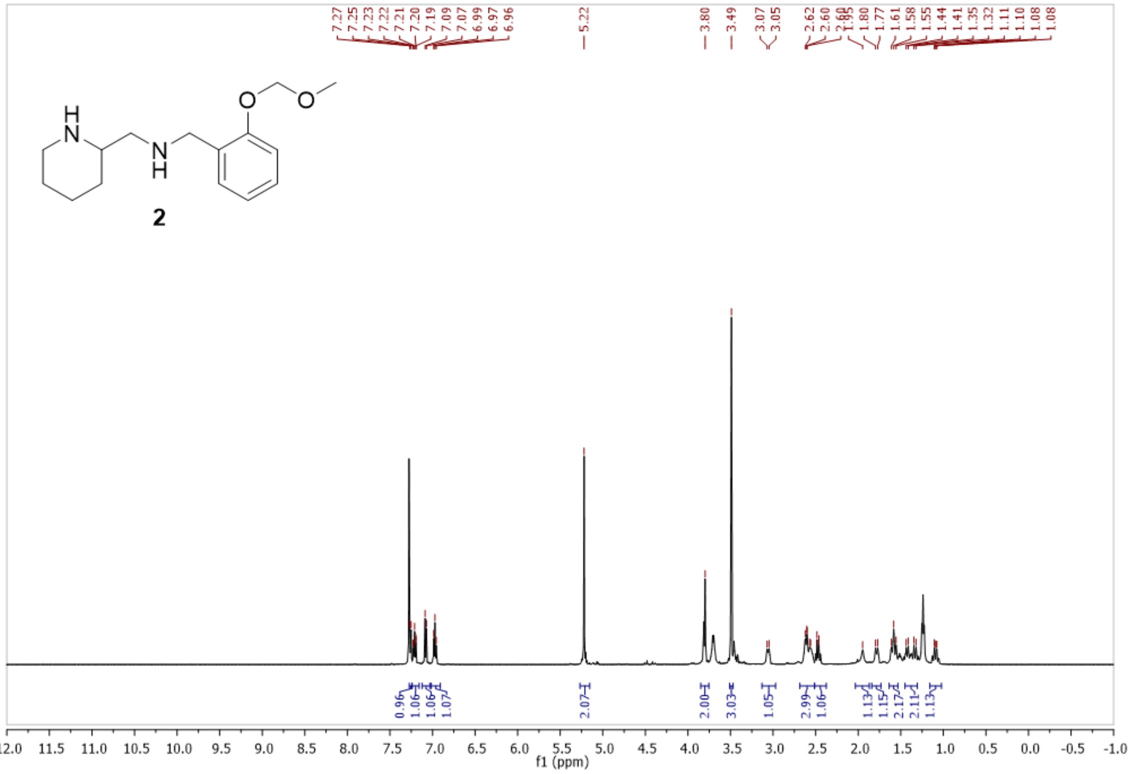


**Figure S3**. $^{1}$H NMR spectrum in $CDCl_3$ of intermediate **2** (signals from residual EtOH at 3.67 and 1.22 ppm are also observable).

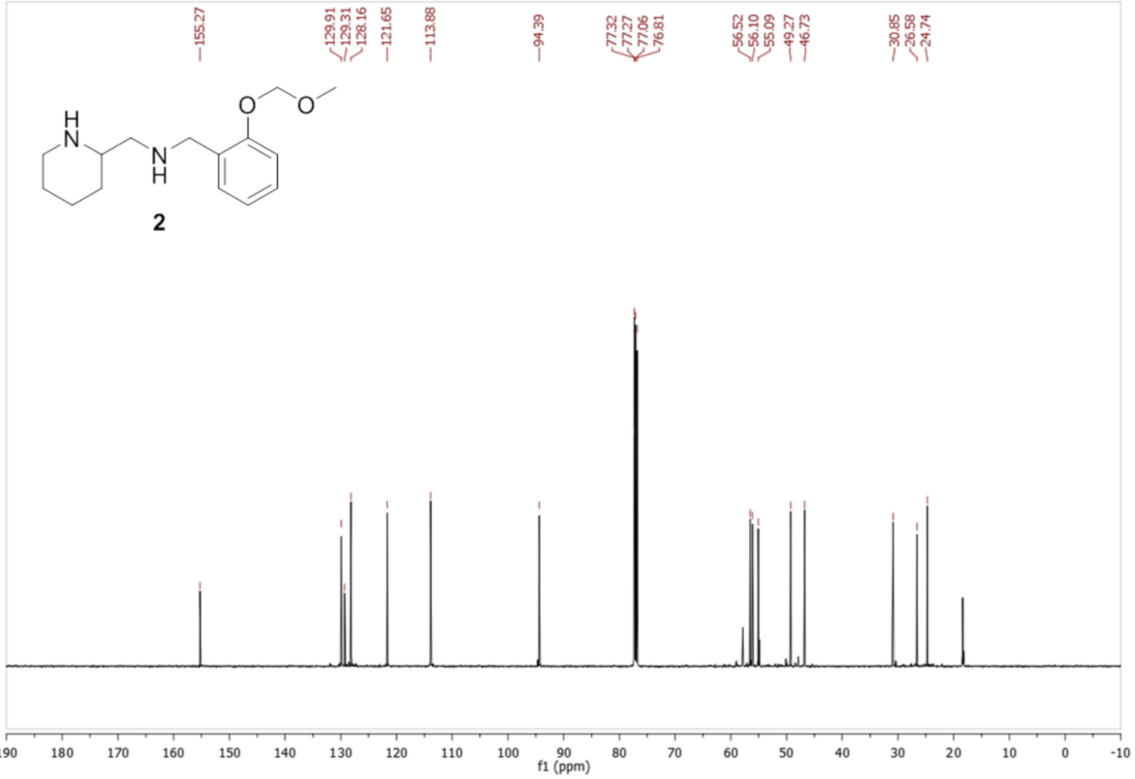


**Figure S4**. $^{13}$C NMR spectrum in $CDCl_3$ of intermediate **2** (signals from residual EtOH at 18.4 and 57.8 ppm are also observable).

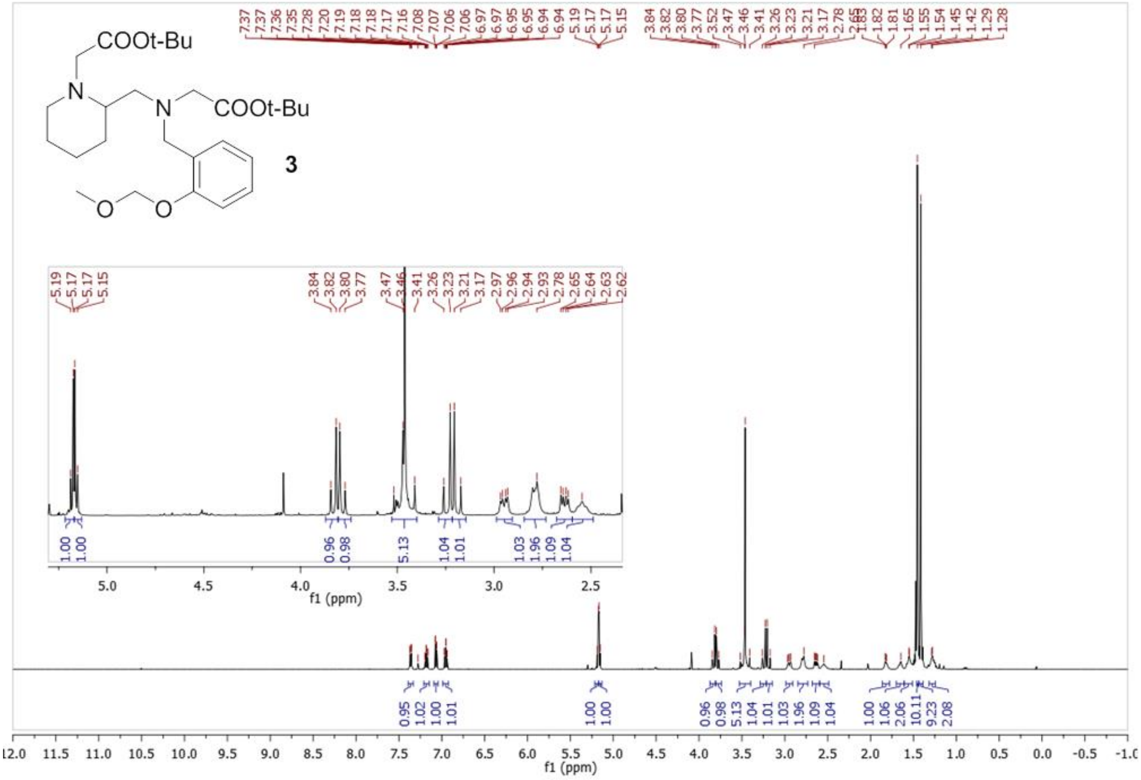


**Figure S5**. $^{1}H$ NMR spectrum in $CDCl_3$ of intermediate **3**.

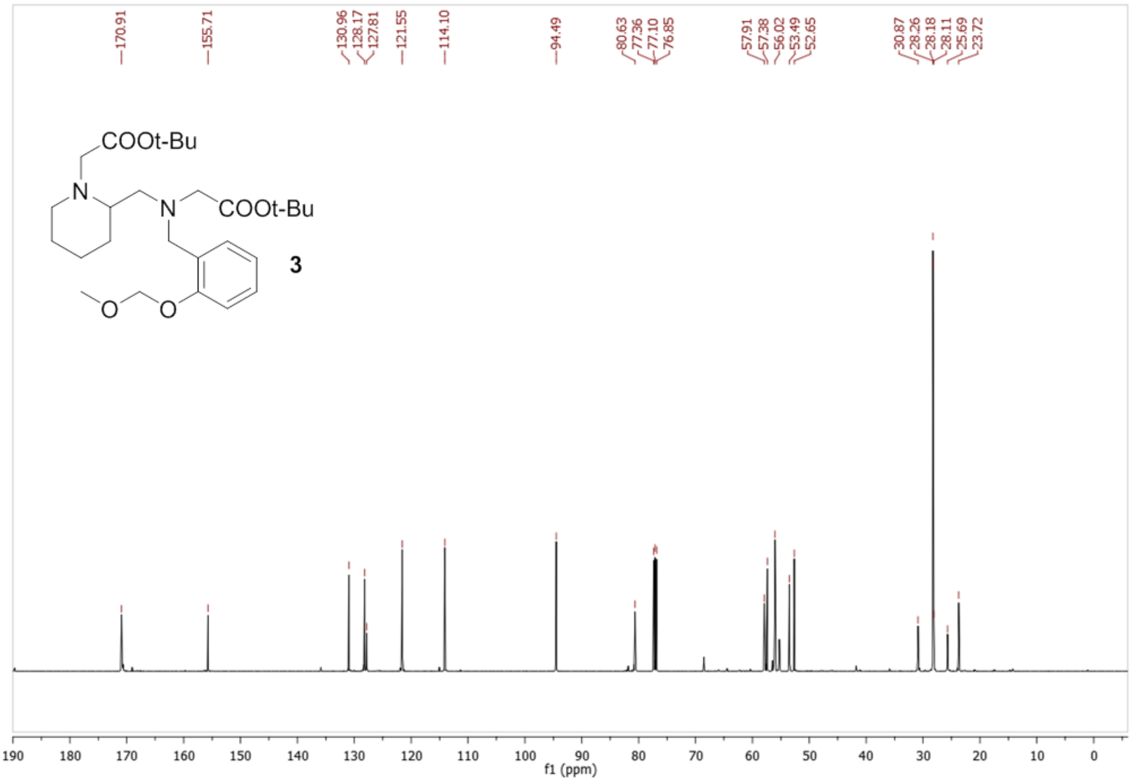


**Figure S6**. $^{13}C$ NMR spectrum in $CDCl_3$ of intermediate **3**.

## 4. HPLC-MS

Analytical HPLC gradient conditions for AMPDA-HB and (*R*)-AMPTA:
Solvent A = $H_2O$ (TFA 0.1%); solvent B = ACN (TFA 0.1%); flow = 1 mL/min;

| Time (min) | Solvent A (%) | Solvent B (%) |
|---|---|---|
| 0 | 99 | 1 |
| 2,00 | 99 | 1 |
| 16,00 | 0 | 100 |
| 19,00 | 0 | 100 |

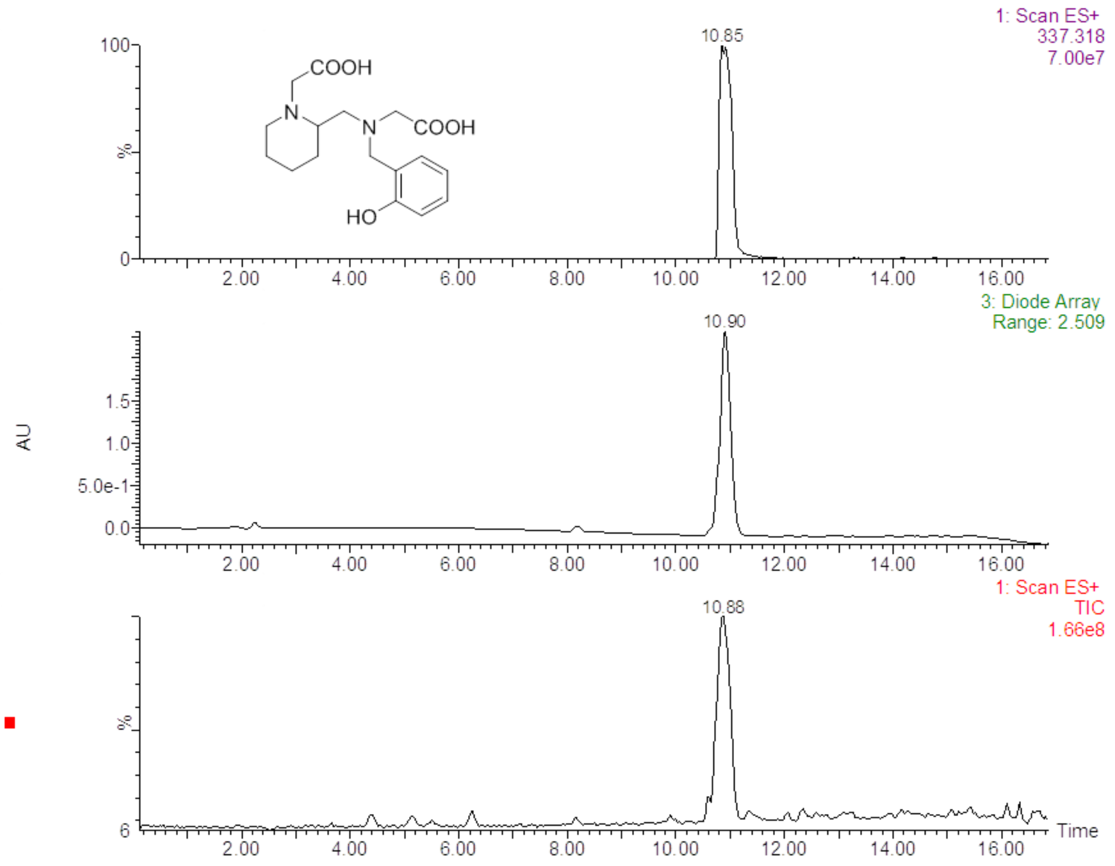


**Figure S7**. ESI$^+$ MS (*bottom*), UV (254 nm, *middle*) and specific ion (M+H$^+$, *top*) HPLC chromatograms of AMPDA-HB ($t_R$ = 10.9 min) prepared according to the new method.

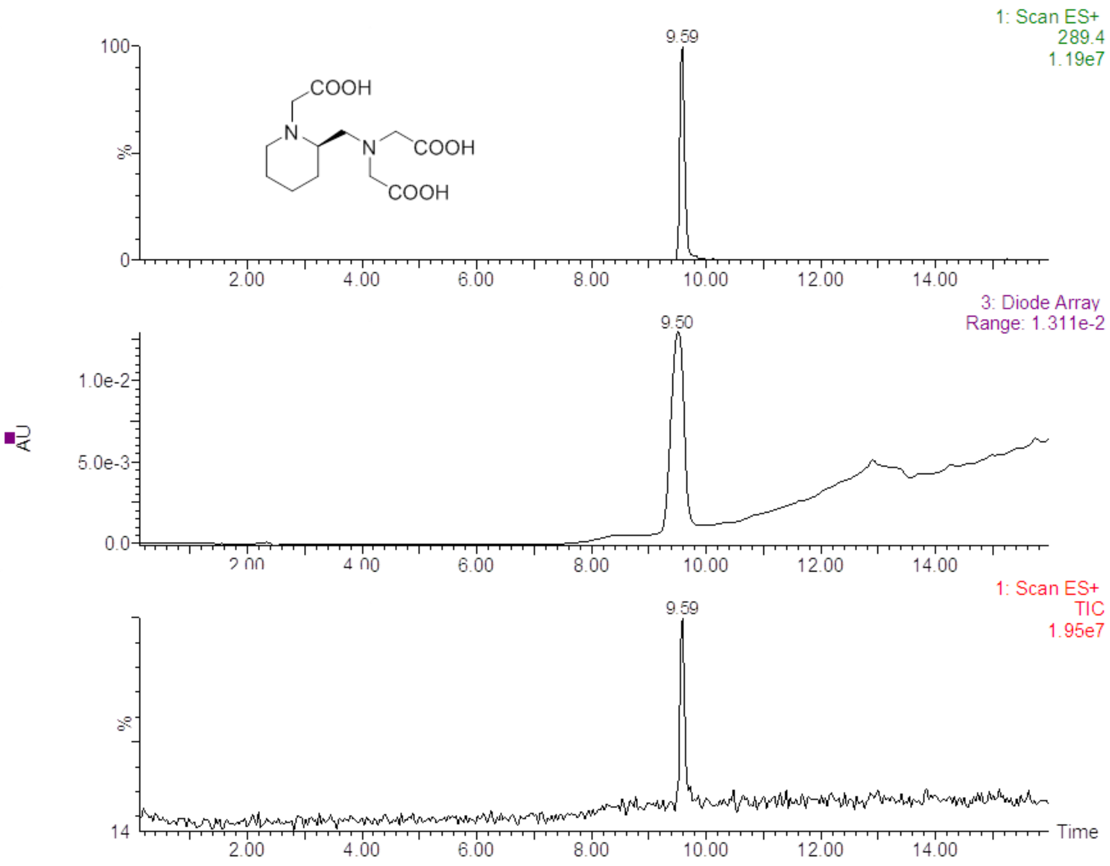


**Figure S8**. ESI$^+$ MS (*bottom*), UV (254 nm, *middle*) and specific ion (M+H$^+$, *top*) HPLC chromatograms of (*R*)-AMPTA ($t_R$ = 9.5 min).

## 5. Equilibrium studies of the Mn(II) complexes

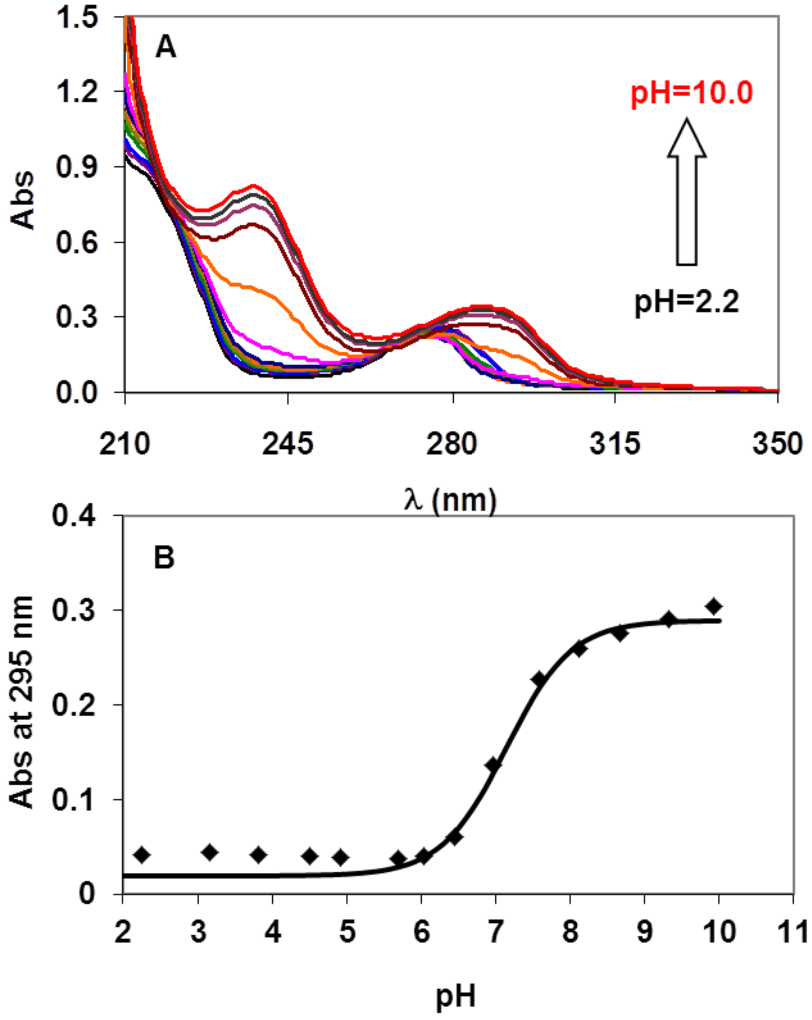


**Figure S9.** Absorption spectra (**A**) and absorbance values at 295 nm (**B**) of $Mn^{2+}$-AMPDA-HB system as a function of pH ($[Mn^{2+}]$ = [AMPDA-HB] = $1.0\times10^{-4}$ M, l = 1.0 cm, 0.15 M NaCl, 25 °C).

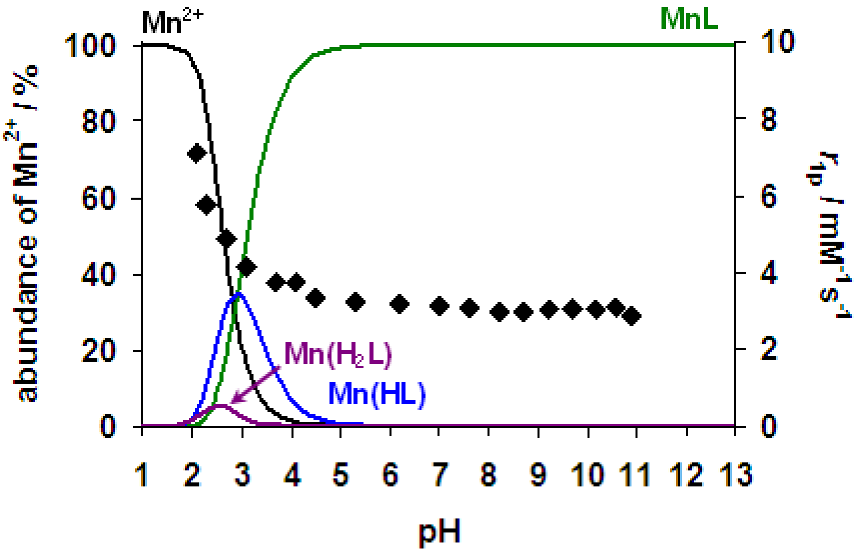


**Figure S10.** Species distribution and relaxivity values (◆) of $Mn^{2+}$-CDTA system as a function of pH ($[Mn^{2+}]$ = [CDTA] = 1.0 mM, 20 MHz, 0.15 M NaCl, 25 ℃).
